# Identification of QTL for kernel weight and size and analysis of the *pentatricopeptide repeat* (*PPR*) gene family in cultivated peanut (*Arachis hypogaea* L.)

**DOI:** 10.1186/s12864-023-09568-y

**Published:** 2023-08-28

**Authors:** Yuanjin Fang, Hua Liu, Li Qin, Feiyan Qi, Ziqi Sun, Jihua Wu, Wenzhao Dong, Bingyan Huang, Xinyou Zhang

**Affiliations:** 1 College of Agriculture, Nanjing Agricultural University, Nanjing, 210095 China; 2 Henan Academy of Agricultural Sciences/Henan Institute of Crop Molecular Breeding/Shennong Laboratory/Key Laboratory of Oil Crops in Huang-Huai-Hai Planis, Ministry of Agriculture and Rural Affairs/Henan Provincial Key Laboratory for Oil Crops Improvement, Zhengzhou, 450002 China; 3 Shangqiu Academy of Agriculture and Forestry, Shangqiu, 476002 China

**Keywords:** Peanut (*Arachis hypogaea* L.), Kernel weight, Kernel size, QTLs (quantitative trait loci), Pentatricopeptide repeat (PPR)

## Abstract

Peanut (*Arachis hypogaea* L.) is an important oilseed crop worldwide. Improving its yield is crucial for sustainable peanut production to meet increasing food and industrial requirements. Deciphering the genetic control underlying peanut kernel weight and size, which are essential components of peanut yield, would facilitate high-yield breeding. A high-density single nucleotide polymorphism (SNP)-based linkage map was constructed using a recombinant inbred lines (RIL) population derived from a cross between the variety Yuanza9102 and a germplasm accession wt09-0023. Kernel weight and size quantitative trait loci (QTLs) were co-localized to a 0.16 Mb interval on Arahy07 using inclusive composite interval mapping (ICIM). Analysis of SNP, and Insertion or Deletion (INDEL) markers in the QTL interval revealed a gene encoding a pentatricopeptide repeat (PPR) superfamily protein as a candidate closely linked with kernel weight and size in cultivated peanut. Examination of the *PPR* gene family indicated a high degree of collinearity of *PPR* genes between *A. hypogaea* and its diploid progenitors, *Arachis duranensis* and *Arachis ipaensis*. The candidate *PPR* gene, *Arahy.JX1V6X*, displayed a constitutive expression pattern in developing seeds. These findings lay a foundation for further fine mapping of QTLs related to kernel weight and size, as well as validation of candidate genes in cultivated peanut.

**Supplementary Information:**

The online version contains supplementary material available at 10.1186/s12864-023-09568-y.

## Introduction

Peanut (*Arachis hypogaea* L.), also known as groundnut, is an essential agricultural commodity consumed globally for its high protein and oil contents. It is cultivated primarily in subtropical and temperate regions and utilized in various forms, including whole nuts, peanut butter, and processed products, such as peanut oil, and the high-protein by-product obtained after oil extraction, which could be used as animal feed. The total global peanut production reached 53.9 million metric tons in 2021 (FAOSTAT https://www.fao.org/faostat/en/#data/QCL). The major peanut-producing countries include China, India, Nigeria, the United States and Sudan. Peanut yield varies among countries and regions, with the United States achieving a yield of 46,352 hg/ha and China reaching 38,706 hg/ha (FAOSTAT 2021). As the world population continues to grow, it is crucial to ensure a stable supply of essential food commodities such as peanuts. Sustainable peanut production plays a vital role in global food security, providing both edible oil and protein for human consumption, as well as by-products for animal feeding. Improving yield is the fundamental goal in peanut breeding.

Pod weight, kernel weight, and shelling percentage are crucial yield components and target traits for yield improvement in peanut breeding. However, these are quantitative traits that are usually coordinated by a large number of genes, typically each with a relatively small effect. The complexity of these traits makes traditional breeding methods challenging and time-consuming. Linkage analysis helps capture the association between phenotype and alleles using high-density markers, such as single nucleotide polymorphism (SNP). By evaluating the effects of these markers in large populations, researchers can identify the genetic basis of these traits and apply molecular markers in breeding programs. Genomics-assisted breeding (GAB) is an essential tool for achieving significant yield improvement in peanut [1]. By implementing GAB, researchers can identify and select specific genes associated with yield components, enabling the development of high-yielding peanut varieties more efficiently than through conventional breeding alone.

Quantitative trait loci (QTLs) associated with yield traits including hundred-pod weight (HPW) and pod size, and hundred-kernel weight (HKW) and kernel size, have recently been identified in peanut. Notably, QTLs for pod size and kernel size, HPW, and HKW were primarily identified on linkage group (LG) A5, A7, and B6, based on genotyping of 188 recombinant inbred lines (RILs) using SSR markers [2]. QTL mapping and genome-wide association study (GWAS) of two nested-association mapping (NAM) populations revealed that genomic regions on chromosomes A05, A06, B05, and B06 were associated with both HPW and HKW in peanut [3]. Moreover, QTLs for kernel size were identified in the 93–102 Mb region on chromosome A05 by SNP array genotyping of a RIL population [4] and on A02 and B06 in 181 RILs genotyped by specific-locus amplified fragment sequencing (SLAF-seq) [5]. A major QTL for kernel weight was identified in the 14.21–17.65 Mb region on chromosome B06, and diagnostic Kompetitive Allele-Specific Polymerase Chain Reaction (KASP) markers were developed [6]. QTL for seed size was fine-mapped on chromosome A07 from 1,148,277 bp to 1,316,744 bp containing 22 annotated genes based on reference sequence of *A. duranensis* [7]. Similarly, pod area and seed weight QTLs were co-localized on chromosome A07 between 0.63 and 1.03 Mbp including 56 genes according to genomic sequence of cultivated peanut ‘Tifrunner’ [8]. Seed size QTL in peanut was investigated by using reference genome of elite founder line ‘Shitouqi’ in China, and the candidate region was located on chromosome 7 containing 99 genes [9]. However, to identify candidate genes, the QTL intervals need to be further refined and narrowed down.

Advancement in sequencing technologies have greatly accelerated genome assembly, and facilitated QTL identification and marker development in various crops, such as rice [10, 11], maize [12], soybean [13, 14], and peanut [9, 15–18]. The availability of whole-genome sequences has provided invaluable resource for studying gene families in these crops. In recent years, genome-wide analyses of various gene families have been conducted in major crops. Notably, several essential gene families, such as *WRKY* and *FAR1*, have been characterized in peanut [19, 20]. Identification and characterization of gene families in peanut will help elucidate their sequence variations and expression patterns under specific condition. In this study, a RIL population was utilized to identify QTLs for kernel traits and to analyze candidate genes for further functional validation. The objectives are to enhance the understanding of the genetic basis of kernel weight and size and to facilitate the improvement of peanut yield traits through molecular breeding.

## Materials and methods

### Plant materials

A RIL population consisting of 521 peanut lines was developed from a cross between Yuanza9102 and wt09-0023. Yuanza9102 is a Spanish-type peanut variety with disease resistance, high oil content and superior yield performance, while wt09-0023 is a high-oleic Runner-type peanut with small seed size (Fig. S1). These RILs were planted in a randomized block design in May across four environments: Yuanyang2021 (2021YY), Shangqiu2021 (2021SQ), Nanyang2021 (2021NY) and Yuanyang2022 (2022YY). Each line was planted in two rows spaced 40 cm apart and 1.5 m long, with 15 cm between plants. Standard management practices were followed, and 450 kg per hectare of compound fertilizer (N:P:K, 14:16:15) was applied before planting. The RILs were harvested in bulked lines in September, and the mature pods for each line were air-dried.

### Phenotyping the RIL population for kernel traits

Twenty mature kernels were selected for each line. Kernel traits, including hundred-kernel weight (HKW), average surface area of the kernel (KA), kernel length (KL), and kernel width (KW), were measured using a seed analyzer SC-G (Hangzhou WanSeen Technology, Hangzhou, China). The phenotypic distributions of HKW, KA, KL and KW were represented as violin plots drawn using Python 3.9 (www.python.org). ANOVA was conducted for each kernel traits in four environments using ANOVA function implemented in QTL IciMapping version 4.2 [21].

### Linkage map construction and QTL identification

A high-density genetic linkage map consisting of 5,120 SNPs was obtained through digested restriction-site associated DNA sequencing (dRAD-seq) of 521 peanut RILs using Tifrunner as reference genome, as previously described by our research team [22]. QTL identification for kernel traits was conducted using the ICIM-ADD model with a step size of 0.1 cM in QTL IciMapping version 4.2 [21]. LG Arahy07 was plotted using MapChart version 2.32 [23]. Variants in the identified QTL interval were selected from our resequencing data, and their effects were predicted using SnpEff version 5.1 [24]. PCR primers were developed for the INDEL marker at 225,751 bp on chromosome Arahy07 by using Primer3 (https://primer3.ut.ee/). Genomic DNA of 31 RILs with extreme HKW across four environments were extracted and diluted to about 50 ng/µL. A 20 µL PCR mix included 4 µL of 5×buffer (Mg^2+^ plus), 1.6 µL of dNTP (2.5 mM), 0.5 µL forward primer (10 µM) and 0.5 µL reverse primer (10 µM), 0.4 µL PrimeSTAR® GXL (Takara Bio Inc.) DNA polymerase, and 3 µL genomic DNA. PCR was performed with initial denaturation at 95℃ for 5 min, followed by 35 cycles of denaturation at 95℃ for 10 s, annealing at 58℃ for 15 s, extension at 68℃ for 1 min 54 s, and a final extension at 68℃ for 7 min. The 1900 bp PCR product were sequenced using forward primer to detect the INDEL genotype of 31 RILs. Primer sequences for the validation panel were available as supplementary file (Table S1).

### Analysis of candidate genes for peanut kernel traits

The protein sequence of *Arahy.JX1V6X* was used to identify PPR family domains (PF13041) from InterPro (https://www.ebi.ac.uk/interpro/). The *PPR* gene family in the Tifrunner genome was identified using HMMER3 (http://www.hmmer.org/), and 685 targets were selected with a cutoff score of 190. Protein sequences of 542 unique genes were extracted using an in-house Python script. The distribution and structure of *PPR* genes were visualized using the gff3 file of the Tifrunner assembly version 1 as input in TBtools version 1.106 [25]. Conserved domains were identified using the NCBI-Conserved Domain Database (CDD) (https://www.ncbi.nlm.nih.gov/Structure/bwrpsb/bwrpsb.cgi), with the e-value threshold set at 1e-15. Multiple alignments were performed using ClustalX 2.1 [26]. A phylogenetic tree was constructed using the maximum parsimony method with 300 bootstrap replicates implemented in MEGA11 [27]. Protein domains and a phylogenetic tree were plotted using the online tool iTOL v6 (https://itol.embl.de/). Syntenic relationships among *A. hypogaea*, *A. duranensis*, *A. ipaensis*, and *Glycine max* were analyzed using the Python JCVI module. Syntenic *PPR* gene pairs were highlighted in different colors in the plot.

### Analysis of *PPR* expression pattern in cultivated peanut Tifrunner

The transcriptome data of Tifrunner were retrieved from the NCBI SRA database, previously deposited under BioProject PRJNA291488 [28]. RSEM [29] was used to map clean data to the Tifrunner genome assembly version 1 [15]. The EBSeq algorithm, implemented in RSEM, was used to identify differentially expressed *PPR* genes at an FDR of 0.05 in five tissues, including leaves, shoots, pegs, pericarps, and seeds collected at different developmental stages. Heatmaps of differentially expressed *PPR* genes were plotted using the “pheatmap” R package. Tissue-specifically expressed *PPR* family members were screened using the tissue specificity (TAU) index, calculated using an in-house Python script. The TAU index ranges from 0 to 1, where a value close to 1 indicates a high degree of tissue-specific expression, while a value close to 0 suggests more ubiquitous or widespread expression across multiple tissues. In this study, *PPR* genes with TAU values greater than 0.7 were identified as being preferentially expressed in specific tissues or conditions.

## Results

### Phenotypic variation of kernel traits in the RIL population

**Fig. 1:**
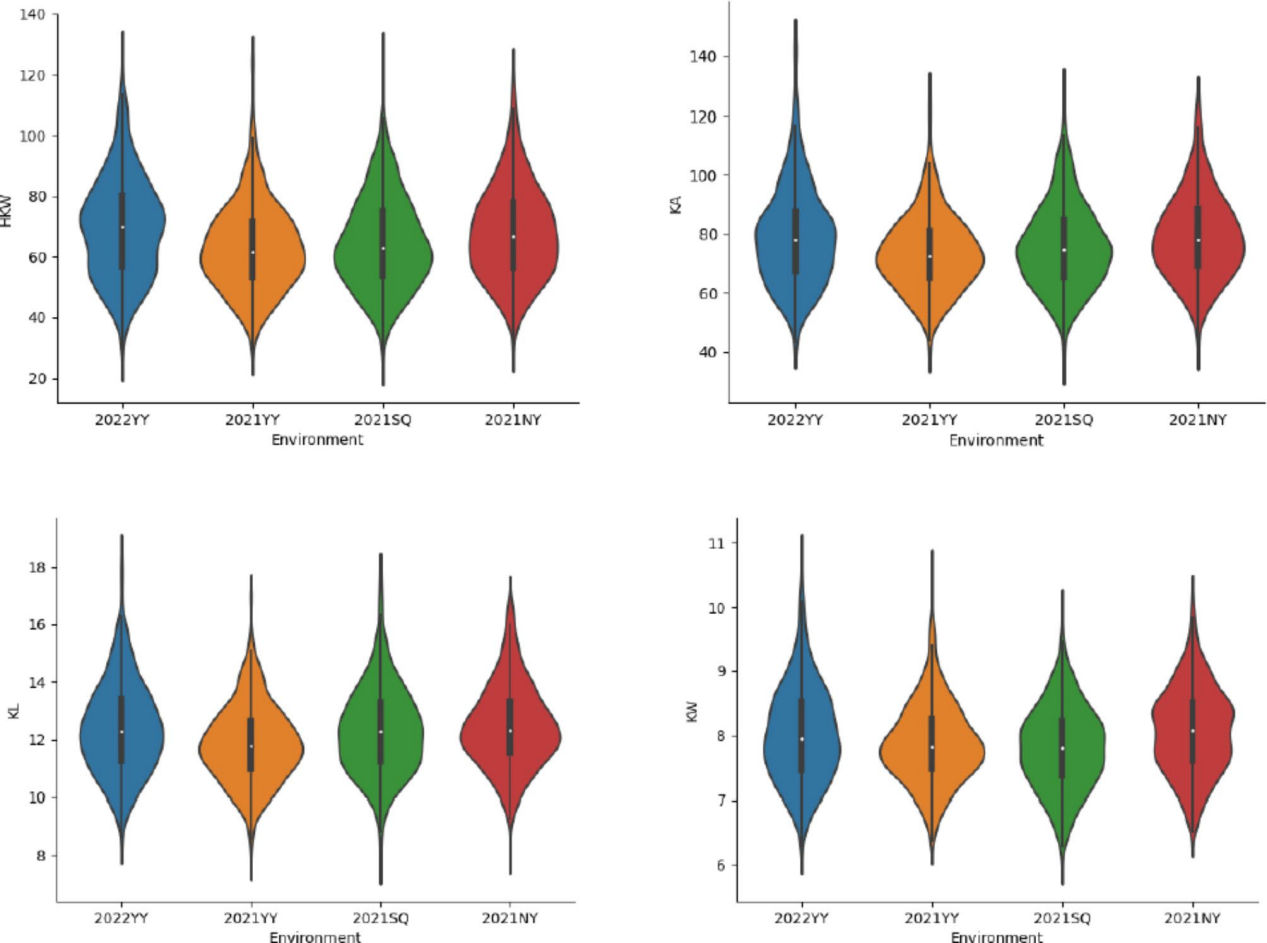
Phenotypic variation of hundred-kernel weight (HKW), kernel area (KA), kernel length (KL), and kernel width (KW) in 521 recombinant inbred lines (RILs) across four different locations. Note: Units for measuring HKW, KA, KL and KW were ‘g’, ‘mm^2^’, ‘mm’ and ‘mm’, respectively

HKW, KA, KL, and KW across four environments were investigated in the RIL population consisting of 521 peanut lines. HKW, KA, KL and KW of parent Yuanza9102 were 77.01 g, 82.17 mm^2^, 12.60 mm and 8.32 mm, averaged over four environments. Kernel weight and size of parent wt09-0023 were smaller than those of Yuanza9102, with averaged HKW 54.33 g, KA 65.74 mm^2^, KL 11.29 mm and KW 7.43 mm. The distribution of each trait at different environments followed a similar pattern (Fig. 1). The HKW ranged from 28.44 to 124.80 g, 29.01 to 124.55 g, 26.19 to 125.36 g, and 30.97 to 119.72 g at environment of 2022YY, 2021YY, 2021SQ and 2021NY, respectively. KA were between 43.77 and 143.21 mm^2^, 40.65 and 127.19 mm^2^, 37.29 and 127.67 mm^2^, 42.31 and 124.82 mm^2^, at 2022YY, 2021YY, 2021SQ and 2021NY, respectively. KL varied from 8.62 to 18.22 mm at 2022YY, 7.90 to 16.98 mm at 2021YY, 7.84 to 17.64 mm at 2021SQ, and 8.17 to 16.85 mm at 2021NY. KW ranged from 6.28 to 10.70 mm at 2022YY, 6.36 to 10.54 mm at 2021YY, 6.06 to 9.91 mm at 2021SQ, 6.51 to 10.11 mm at 2021NY. Significant positive correlations between HKW, KA, KL, and KW were revealed across all four environments (Fig. S2-S5). Significant difference was observed for each kernel trait in the RIL population and among four environments (Table S2).

### Identification and validation of QTLs for kernel traits in peanut

**Fig. 2:**
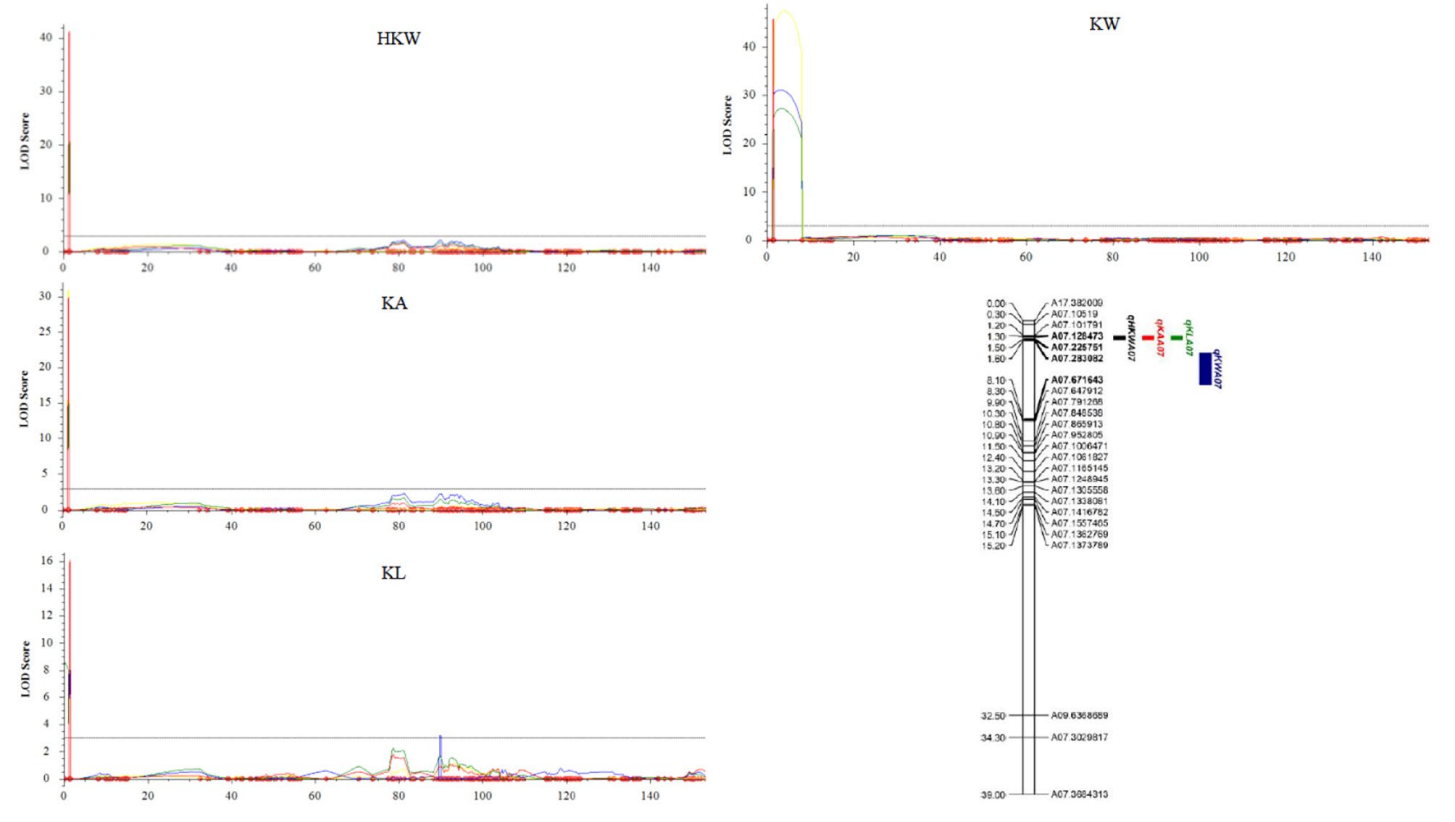
QTLs associated with hundred kernel weight (HKW), kernel area (KA), kernel length (KL) and kernel width (KW) identified in LG 7 in 521 recombinant inbred lines (RILs) across four different environments (orange line: Yuanyang in 2022, green line: Yuanyang in 2021, blue line: Shangqiu in 2021, yellow line: Nanyang in 2021)

QTLs for HKW, KA, and KL were identified in a region spanning 1.25 cM to 1.55 cM on LG 7 in at least three distinct locations (Fig. 2). Both *qHKWA07* and *qKAA07* were flanked by markers A07.128473 and A07.283082, with LOD (likelihood of the odds) scores ranging from 17.38 to 41.22 and a PVE (phenotypic variance explained) value exceeding 10% (Table 1). QTL for kernel length, *qKLA07*, was co-localized in the same interval as *qHKWA07* and *qKAA07*, and the LOD scores were between 12.36 and 16.10 with PVE ranging from 10.21 to 14.21 (Table 1). QTL for KW was located between 2.65 cM and 5.25 cM on LG 7 (Fig. 2), among which *qKWA07* was identified in the interval flanked by markers A07.283082 and A07.671643. For this QTL, the LOD scores were between 27.23 and 47.42, and the PVE values were greater than 20% (Table 1). The positive additive effects for *qHKWA07*, *qKAA07*, *qKLA07*, and *qKWA07* indicate that alleles from Yuanza9102 contributed to the larger seed size observed in the RIL population. The co-localized QTL intervals for HKW, KA, and KL on chromosome Arahy07 contain 25 predicted genes among which 13 encode PPR (Table S3). By leveraging the resequencing data of the RIL parents, a polymorphic INDEL was identified at 225,751 bp located in the coding region of *Arahy.JX1V6X*, which encodes a PPR superfamily protein (Table 2). This INDEL was predicted to lead to a frameshift mutation of *Arahy.JX1V6X.* Furthermore, 52 annotated genes were included in the QTL region for kernel width (Table S4). A SNP associated with *qKWA07* was identified at 321,806 bp on chromosome Arahy07. This marker was located in the coding sequence of *Arahy.P2ZS9F*, which also encoded a PPR superfamily protein, and could lead to a missense mutation. Besides, missense SNP variants in the coding sequences of *Arahy.LFBK1H* (annotated as heat shock protein) and *Arahy.VAAE0N* (predicted ATP-binding protein) were identified in the interval of *qKWA07* by using our resequencing data of two RIL parents. The INDEL marker was validated in 31 RILs including 19 RILs with small HKW and 12 RILs with large HKW. The INDEL genotype “GC”, the same genotype as the parent Yuanza9102, was shown to be closely linked with large HKW, while the “G” genotype from parent wt09-0023 was linked with small HKW (Table 3).

**Table 1:** QTLs for HKW, KA, KL, and KW identified in the RIL population derived from the cross between Yuanza9102 and wt09-0023

QTL	Linkage group	Position (cM)	Marker Interval	LOD	PVE(%)	Additive effect	Environment
*qHKWA07*, *qKAA07*	7	1.25 to 1.55	A07.128473 - A07.283082	17.38 to 41.22	14.82 to 31.08	4.87 to 9.23	2022YY, 2021YY, 2021SQ, 2021NY
*qKWA07*	7	2.65 to 5.25	A07.283082 - A07.671643	27.23 to 47.42	21.97 to 36.58	0.30 to 0.42	2021YY, 2021SQ, 2021NY
*qKLA07*	7	1.25 to 1.55	A07.128473 - A07.283082	12.36 to 16.10	10.21 to 14.21	0.48 to 0.58	2022YY, 2021SQ, 2021NY

**Table 2:** Candidate genes for hundred-kernel weight (HKW), kernel area (KA), kernel length (KL), and kernel width (KW) on chromosome Arahy07

QTL	Marker Position	Marker Type	Predicted Effect	Gene Annotation	
*qHKWA07* *qKAA07* *qKLA07*	Arahy07.225751	INDEL	frameshift; high effect	arahy.Tifrunner.gnm1.ann1.JX1V6X.1	Pentatricopeptide repeat (PPR) superfamily protein
*qKWA07*	Arahy07.321806	SNP	missense; moderate effect	arahy.Tifrunner.gnm1.ann1.P2ZS9F.1	Pentatricopeptide repeat (PPR) superfamily protein

**Table 3:** Validation of the INDEL marker in 31 RILs with extreme HKW

RIL	Marker genotype	HKW_SQ2021	HKW_NY2021	HKW_YY2021	HKW_YY2022
P265	C	59.66	58.71	42.00	65.98
P270	“-“	52.26	52.10	44.40	48.74
P280	“-“	26.19	30.97	29.01	28.44
P309	“-“	44.83	47.32	41.76	49.74
P322	“-“	52.59	53.25	44.46	53.67
P324	“-“	34.87	NA	36.09	33.15
P348	“-“	44.71	47.82	39.40	47.84
P370	“-“	45.77	46.11	41.91	48.15
P409	“-“	49.58	50.19	45.31	48.74
P421	“-“	44.59	NA	36.70	44.32
P439	C	44.33	43.64	42.21	43.87
P446	C	44.42	63.65	44.00	51.07
P535	“-“	44.16	47.76	43.32	42.66
P538	“-“	46.97	44.06	45.30	42.76
P544	“-“	46.75	44.34	45.25	50.56
P567	“-“	46.92	49.41	45.74	46.91
P593	C	55.38	72.71	43.73	68.95
P623	“-“	42.00	40.70	39.61	41.03
P754	“-“	40.08	44.83	44.08	44.96
P278	C	94.93	95.66	84.64	80.58
P296	C	125.36	113.62	101.43	115.63
P362	“-“	NA	82.16	87.38	79.44
P364	C	80.99	93.23	90.68	107.97
P365	C	51.93	68.29	86.32	89.45
P456	“-“	95.70	NA	89.46	83.69
P504	C	90.11	102.78	111.32	107.31
P602	C	89.86	92.64	NA	104.78
P612	C	82.20	99.47	86.50	86.90
P672	C	85.05	83.69	95.22	96.30
P681	C	71.33	96.62	99.08	92.09
P795	C	82.08	74.25	87.82	93.15
P798 (Yuanza9102)	C	72.44	76.77	75.20	83.64
P799 (wt09-0023)	“-“	50.93	56.21	54.92	55.26

### Characteristics of the *PPR* gene family in the cultivated peanut genome

**Fig. 3:**
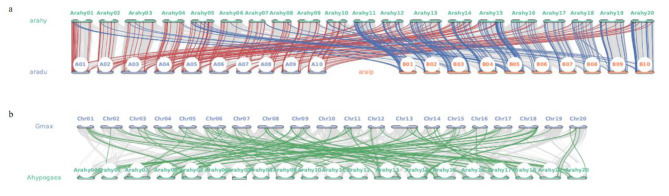
Synteny plots between *Arachis hypogaea* and its diploid progenitors *Arachis duranensis* and *Arachis ipaensis***(a)** and between *A. hypogaea* and *Glycine max***(b)**. Red line represents the *PPR* genes in colinear blocks between *A. hypogaea* and *A. duranensis*. Blue line represents the *PPR* genes in colinear blocks between *A. hypogaea* and *A. ipaensis*. Green line represents the *PPR* genes in colinear blocks between *A. hypogaea* and *G. max*

The *PPR* gene family, comprising 542 unique genes, was identified in the Tifrunner genome. They were found at 20 chromosomal ends within the Tifrunner genome (Fig. S6). A number of structural features of the *PPR* genes were observed based on the Tifrunner genome annotation (Fig. S7). For example, the coding sequences of both *Arahy.JX1V6X* and *Arahy.P2ZS9F* consist of a single exon, whereas *Arahy.9W1T0L* and *Arahy.SH1YAP* each contain three exons in their respective coding sequences. Phylogenetic analysis revealed that these PPR protein sequences could be classified into three groups based on distinct domain structures (Fig. S8). PPR proteins with the PPR_2 domain were closely clustered with proteins containing the PLN03218 domain. The DYW motif was a unique feature of the group identified with the PLN03077 domain. Specifically, *Arahy.JX1V6X* was identified in a group containing the conserved PLN03218 domain in protein sequences, while *Arahy.P2ZS9F* belonged to a cluster characterized by PLN03077 domain and DYW motif (Fig. S8). Synteny analysis between cultivated peanut Tifrunner and its diploid progenitors *A. duranensis* and *A. ipaensis* led to the identification of 170 and 187 orthologous *PPR* gene pairs, respectively (Fig. 3a). Interestingly, approximately 35% of Tifrunner *PPR* genes in syntenic pairs with genes of *A. duranensis* also exhibited a colinear relationship with *A. ipaensis* genes (Table S5). Moreover, about 32% of Tifrunner *PPRs* paired with *A. ipaensis* were identified in colinear blocks between Tifrunner and *A. duranensis*. A more complex syntenic relationship was observed between *A. hypogaea* and *G. max* (Fig. 3b). One hundred and thirteen *PPR* gene pairs were identified in the colinear blocks between *A. hypogaea* and *G. max*, suggesting a close evolutionary relationship between these species.

### Expression pattern of *PPR* genes in Tifrunner

The *PPR* genes with differential expression across five tissue types at various developmental stages were identified, and their relative expression was presented using a heatmap (Fig. S9). Out of the 466 differentially expressed *PPR* genes, 56 exhibited tissue-preferred expression patterns (Table S6). Most of these *PPR* genes displayed leaf-specific expression, while *Arahy.51T3ZK*, *Arahy.8QK61L*, and *Arahy.8HC1KC* showed preferential expression in seeds. In contrast, *Arahy.JX1V6X* and *Arahy.P2ZS9F* did not show tissue-specific expression patterns. An investigation of seeds at four developmental stages revealed 134 differentially expressed *PPR* genes (Fig. S10). The majority of these genes demonstrated increased expression levels from seed pattee stage six to seed pattee stage ten. The expression levels of 110 differentially expressed *PPR* genes were highest in seeds among five tissue types (Table S7). Furthermore, 42 of these 110 *PPR* genes exhibited differential expression pattern throughout four stages of developing seed (Fig. S10).

## Discussion

Kernel weight and size are important yield traits in peanut, governed by multiple genes and controlled by regulatory networks during seed development. It was previously shown that QTLs for HKW and kernel size were co-localized on chromosomes 2 and 16 in a RIL population derived from Huayu36 and a breeding line ‘6–13’ [5]. They found that three candidate genes involved in the brassinosteroid (BR) pathway and a gene encoding an auxin response factor 2 (ARF2)-like protein were identified in the stable QTL regions on chromosome 2 and chromosome 16, respectively. Kernel weight and pod weight QTLs were co-localized in the 93–102 Mb region on LG A05, with a LOD score exceeding 19 and PVE greater than 36% [4]. Joint inclusive composite interval mapping (JICIM) and GWAS for two nested-association mapping (NAM) populations revealed co-localization of seed and pod weight QTLs on chromosomes A05, A06, B05, and B06 [3]. And candidate genes, including a *PPR* gene, *Aradu.217QF*, were identified by significant associations between SNP markers and pod or seed weight. In another study of QTL for kernel weight, *qSWB06.3* was identified in an interval of 2.07 Mb on chromosome B06 [6]. Meanwhile, candidate genes AH16G10100 (homologous to a gene controlling seed size in *Arabidopsis*) and AH16G09300 (homologous to a gene regulating grain size in rice) located in *qSWB06.3* were inferred from RNA-seq analysis of the parents of the RILs at different stages of seed development. QTLs for seed and pod traits were co-localized on B06 and B07 based on reference sequence from two progenitors of cultivate peanut [30], indicating strong genetic association between pod and kernel traits. GWAS of 250 accessions from Chinese peanut mini-core collection revealed significant associations between pod and kernel traits and SNP loci on chromosomes A06 and A02 [31]. Fine-mapping of pod size QTL narrowed the interval to 36.46 kb on chromosome Arahy07 [32], which overlapped with *qKWA07* in this study. In the present study, using a RIL population derived from Yuanza9102 and wt09-0023, three stable QTLs, *qHKWA07* ,*qKAA07* and *qKLA07*, were co-localized in a 0.16 Mbp interval on chromosome Arahy07, with the highest PVE reaching 31.08%. The co-localized intervals of *qHKWA07*, *qKAA07*, and *qKLA07* contained 25 predicted genes with 13 genes encoding PPR (Table S3), while the interval of *qKWA07* included 52 annotated genes (Table S4). By selecting polymorphic markers between RIL parents in our resequencing data and predicting their impact on gene functions, an INDEL in the coding sequence of *Arahy.JX1V6X* and a SNP in the coding sequence of *Arahy.P2ZS9F* were both polymorphic between the RIL parents and predicted to have moderate or high impact on functions of these genes (Table 2). Meanwhile, the INDEL and SNP variants closely linked with kernel traits were not likely due to genetic structure from two subspecies of cultivated peanut as indicated by a recent study [33]. The INDEL marker was validated by PCR in 31 RILs and was closely linked with HKW (Table 3). As a quantitative trait, candidate genes for HKW and kernel size were identified on chromosomes 2, 5, 7 and 16 in populations derived from different genetic background [3, 5, 6, 32]. In this study, a *PPR* gene on chromosome Arahy07 were identified for HKW and KA, which would contribute to understanding the genetic control of kernel weight and size in cultivated peanut.

PPR comprises tandem arrays of a 35-amino-acid motif, which is commonly found in the eukaryotic genome. The *PPR* genes were first discovered in *Arabidopsis* and described by Small and Peeters [34]. They are abundant in plant genomes and have been shown to encode proteins localized to mitochondria and chloroplasts [35, 36]. In *Arabidopsis thaliana*, 441 *PPR* genes were identified [37], while 521 *PPR* genes were found in maize, where they were found to be involved in organelle function and stress response [38]. PPR proteins can be classified to P subfamily and PLS subfamily, based on their motif structure [37]. In the present study, the *PPR* gene *Arahy.P2ZS9F* was identified in the *qKWA07* interval, and its protein sequence contained a DYW C-terminal motif, recognized as a member of the PLS subfamily (Fig. S8). In addition, RNA-seq data analysis from Tifrunner indicated that *Arahy.P2ZS9F* was differentially expressed among five different tissue types and at different stages of seed development (Fig. S9 and S10). The candidate gene *Arahy.JX1V6X* was identified in the co-localized interval of *qHKWA07* and *qKAA07* and exhibited a constitutive expression pattern in developing seeds.

In rice, the *ppr5* mutant showed significantly lower thousand-grain weight than the wild type, and it was found that PPR5 is responsible for splicing *nad4* intron 3 in mitochondria and endosperm development [39]. Over 400 *PPR* genes were identified in the B73 and PH207 maize genomes [40]. Furthermore, expression data from maize kernels revealed significant correlations between *PPR* genes and HKW and KW [40]. Studies of *ppr* mutants in maize, rice, and Arabidopsis revealed their roles in RNA metabolism including RNA cleavage, RNA degradation, and RNA stability [36]. A maize *PPR* mutant, *Zmsmk9*, was identified by map-based cloning and was shown to be responsible for decreased splicing efficiency of mitochondrial *nad5* intron-1 and intron-4 [41]. In rice, *PPR939* was found to be involved in plant growth and pollen development. The studies on the rice mutant *osppr939*, produced by CRISPR/Cas9, revealed the role of *OsPPR939* in splicing mitochondrial *nad5* introns 1, 2, and 3 [42]. Our grasp of PPR functions in peanuts remains incomplete due to the paucity of studies in this area. Transcriptome analyses of developing seeds from two accessions of the cultivated peanut and the wild tetraploid species *Arachis monticola* indicated an association between a candidate gene encoding PPR protein and seed size/weight on chromosome A05 of *A. monticola* genome [43]. Interestingly, a major QTL for shelling percentage in peanut *qSPA07.1* was identified on chromosome A07 in a 0.73 Mb-interval containing *Arahy.JX1V6X* and *Arahy.P2ZS9F* as candidate genes [44]. In addition, a major QTL for pod weight and size was identified on chromosome A07 from 0.06 to 1.54 Mb containing 147 annotated genes among which *Aradu.50R4M*, *Aradu.VVK9N*, and *Aradu.HUV25* were putative *PPR* genes [45]. Moreover, *Aradu.HUV25* was paired with *Arahy.P2ZS9F* in a collinear block identified between Tifrunner (*A. hypogaea*) and *A. duranensis* (Fig. 3a). In this study, two *PPR* genes, *Arahy.JX1V6X* and *Arahy.P2ZS9F*, were identified in the QTL intervals linked with kernel weight and size and were differentially expressed across different developmental stages in the peanut cultivar Tifrunner, implying their association with plant growth and kernel development in cultivated peanut.

To conclude, this study identified a major QTL closely linked with kernel traits in peanut and explored the expression profile and gene family of the candidate *PPR* genes. *qHKWA07* and *qKAA07* could be utilized as a potential target in future molecular breeding strategies to improve yield traits. The candidate *PPR* gene *Arahy.JX1V6X* on Arahy07 will be validated via over-expression and gene editing techniques in peanut, in order to uncover its role associated with kernel weight and size.

### Electronic supplementary material

Below is the link to the electronic supplementary material.

**Additional file 1:** Supplementary Tables S1-S7.

**Additional file 2:** Fig S1. Kernels of the RIL parents Yuanza9102 and wt09-0023.

**Additional file 3:** Fig S2. Correlation between HKW, KA, KL and KW in Yuanyang 2021.

**Additional file 4:** Fig S3. Correlation between HKW, KA, KL and KW in Shangqiu 2021.

**Additional file 5:** Fig S4. Correlation between HKW, KA, KL and KW in Nanyang 2021.

**Additional file 6:** Fig S5. Correlation between HKW, KA, KL and KW in Yuanyang 2022.

**Additional file 7:** Fig S6. Distribution of the *Pentatricopeptide Repeat* (*PPR*) gene family in the *Arachis hypogaea* cv Tifrunner genome.

**Additional file 8:** Fig S7. Gene structure of the *Pentatricopeptide Repeat* (*PPR*) genes in the cultivated peanut (*Arachis hypogaea* L.).

**Additional file 9:** Fig S8. Phylogenetic tree and conserved domains of the *Pentatricopeptide Repeat* (*PPR*) gene family in peanut.

**Additional file 10:** Fig S9. Heatmap of the differentially expressed *PPR* genes across five different tissues of Tifrunner including leaf, shoot, peg, pericarp, and seed.

**Additional file 11:** Fig S10. Heatmap of the differentially expressed *PPR* genes across four different stages of seed development in Tifrunner.

## Data Availability

The data generated and analyzed in this study are included in the manuscript and supplementary files.
