# Supplementary figures and images for: Identification of QTL for kernel weight and size and analysis of the pentatricopeptide repeat (PPR) gene family in cultivated peanut (Arachis hypogaea L.)
Source: BMC Genomics. 2023 Aug 28;24:495. doi: 10.1186/s12864-023-09568-y (PMC10463326; doi:10.1186/s12864-023-09568-y)

**Fig. S1** Kernels of the RIL parents Yuanza9102 (bottom) and wt09-0023 (top)

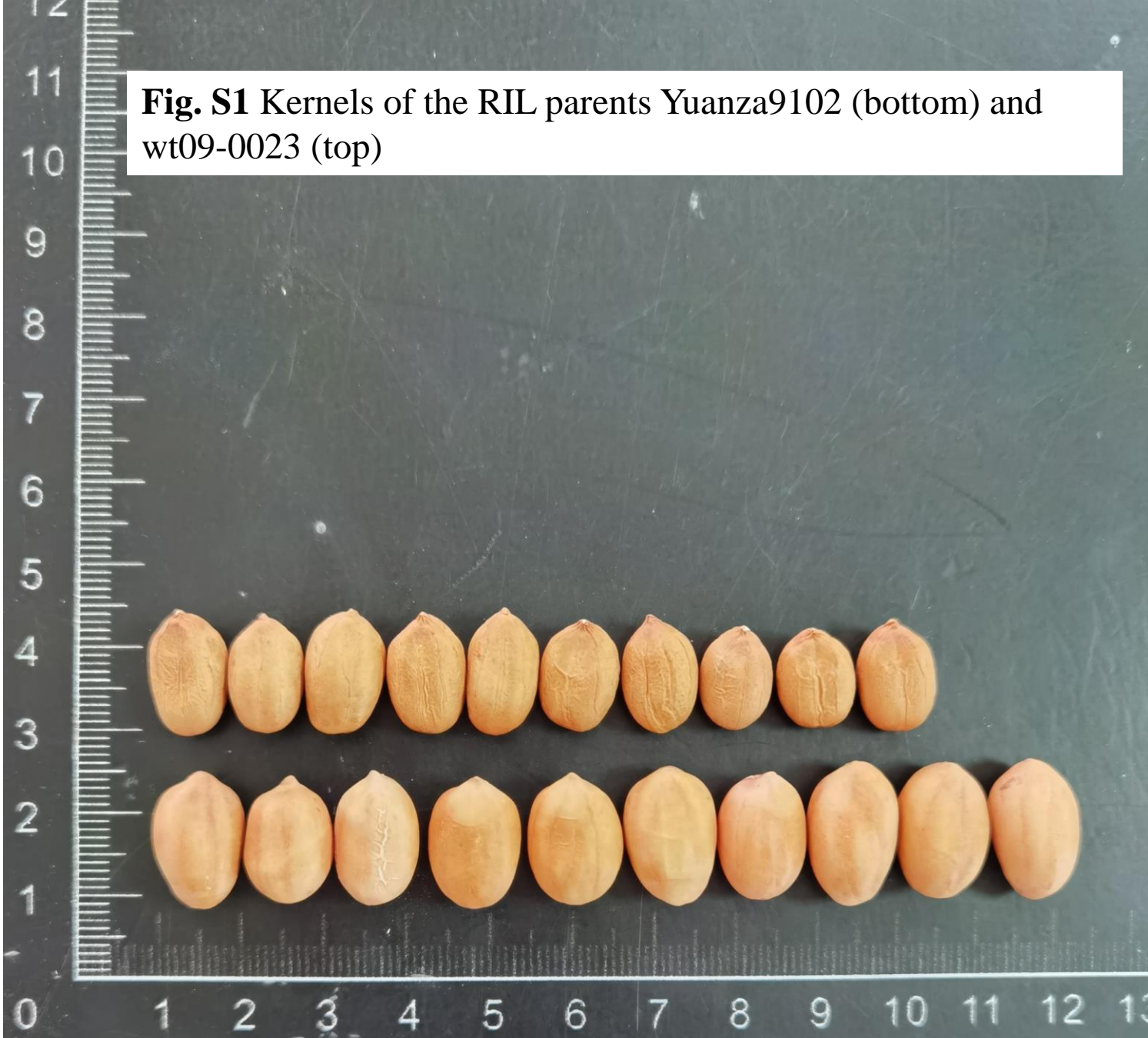

Note: measured in cm

Supplement: Supplementary file 2 — Additional file 2: Fig S1. Kernels of the RIL parents Yuanza9102 and wt09-0023. [file 12864_2023_9568_MOESM2_ESM.pdf]

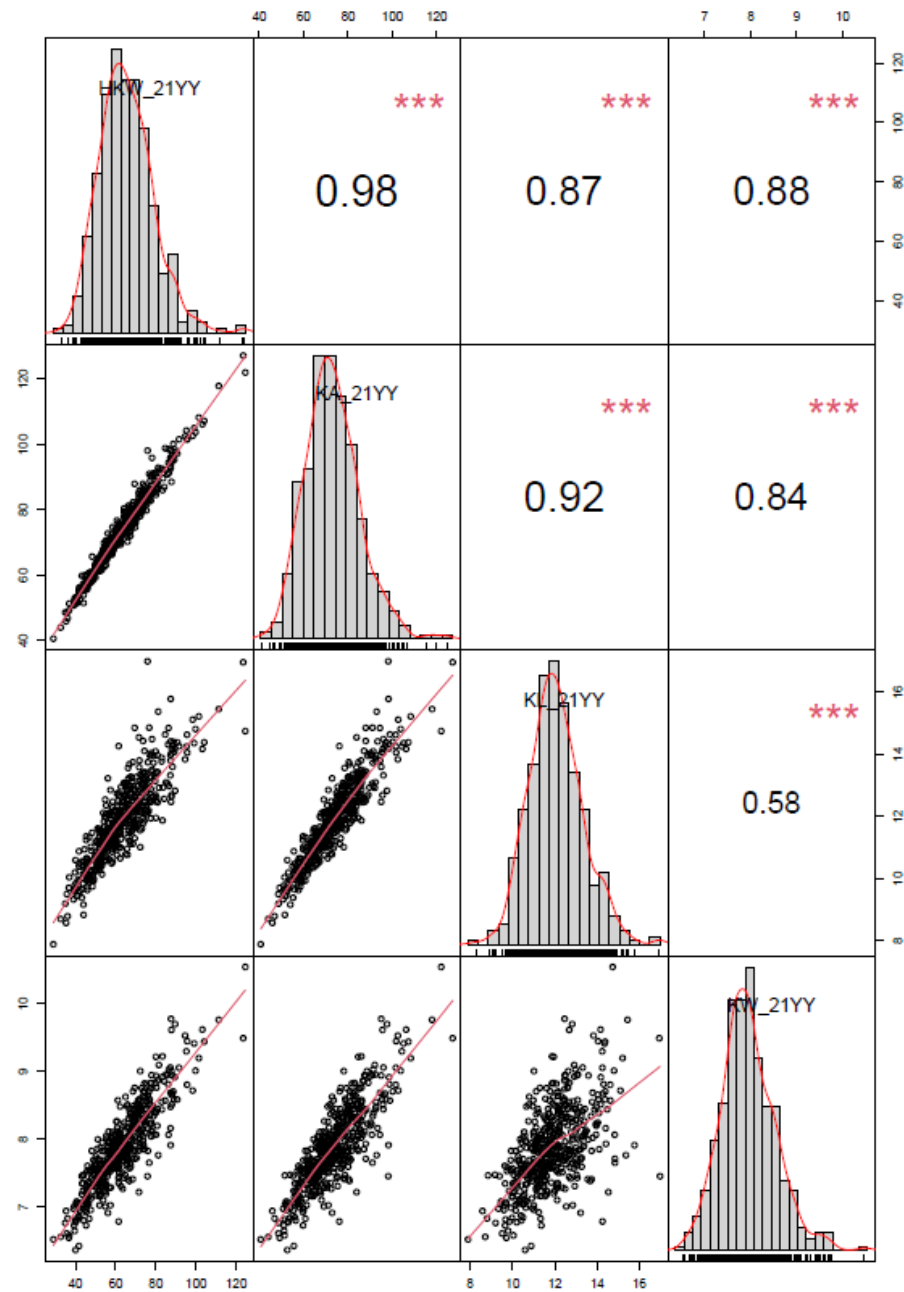

**Fig. S2** Correlation between HKW, KA, KL and KW in Yuanyang 2021.

Supplement: Supplementary file 3 — Additional file 3: Fig S2. Correlation between HKW, KA, KL and KW in Yuanyang 2021. [file 12864_2023_9568_MOESM3_ESM.pdf]

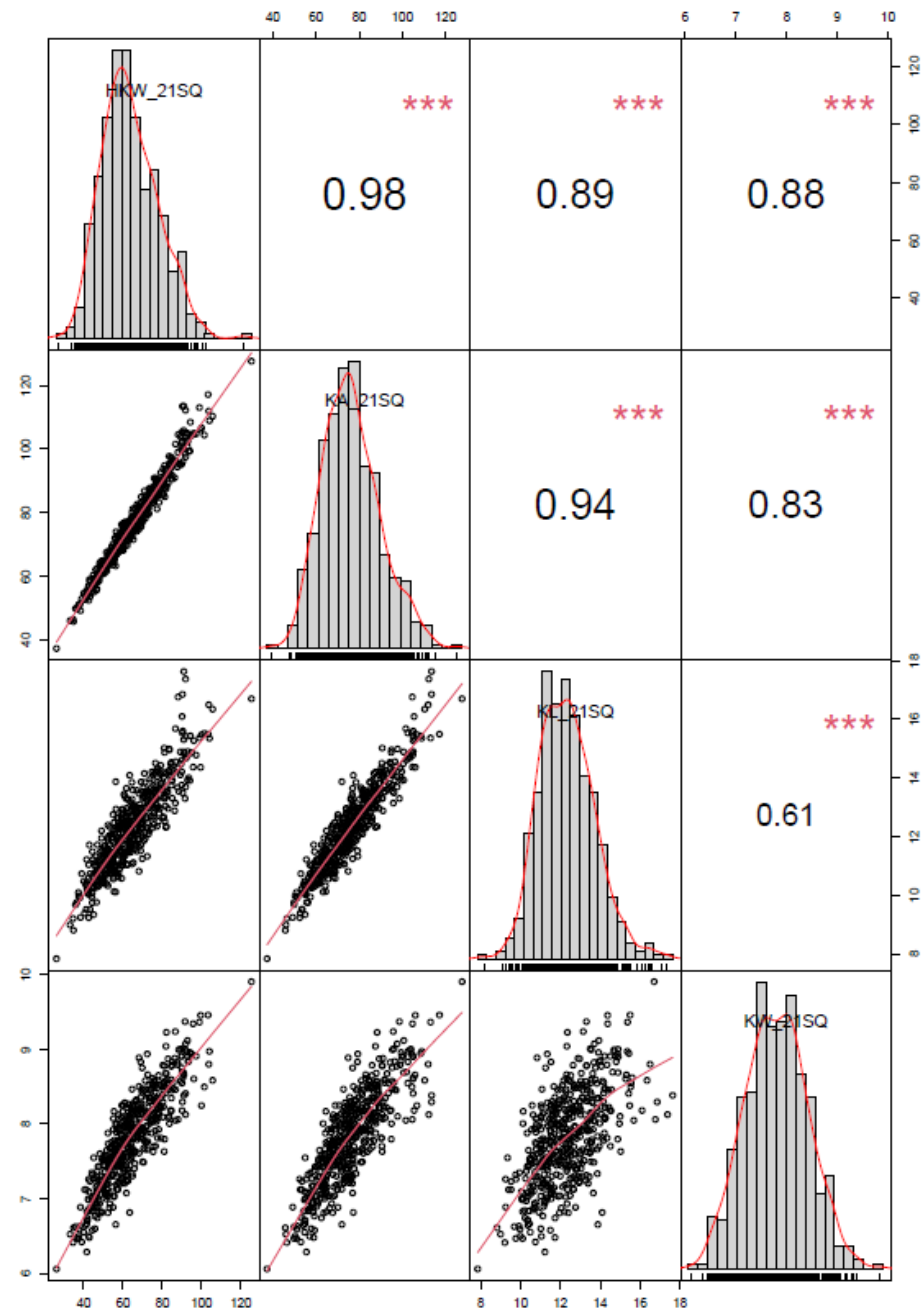

**Fig. S3** Correlation between HKW, KA, KL and KW in Shangqiu 2021.

Supplement: Supplementary file 4 — Additional file 4: Fig S3. Correlation between HKW, KA, KL and KW in Shangqiu 2021. [file 12864_2023_9568_MOESM4_ESM.pdf]

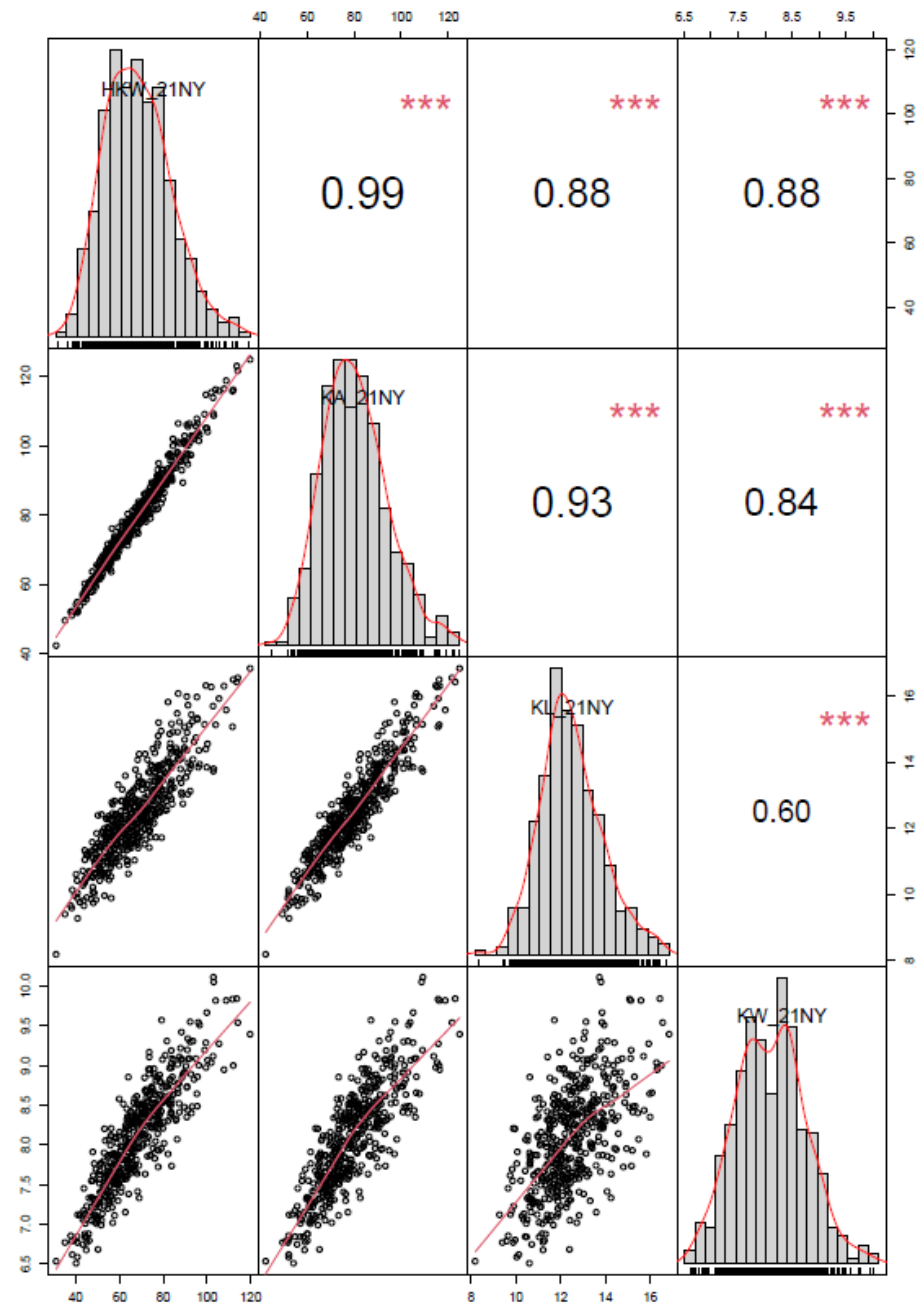

**Fig. S4** Correlation between HKW, KA, KL and KW in Nanyang 2021.

Supplement: Supplementary file 5 — Additional file 5: Fig S4. Correlation between HKW, KA, KL and KW in Nanyang 2021. [file 12864_2023_9568_MOESM5_ESM.pdf]

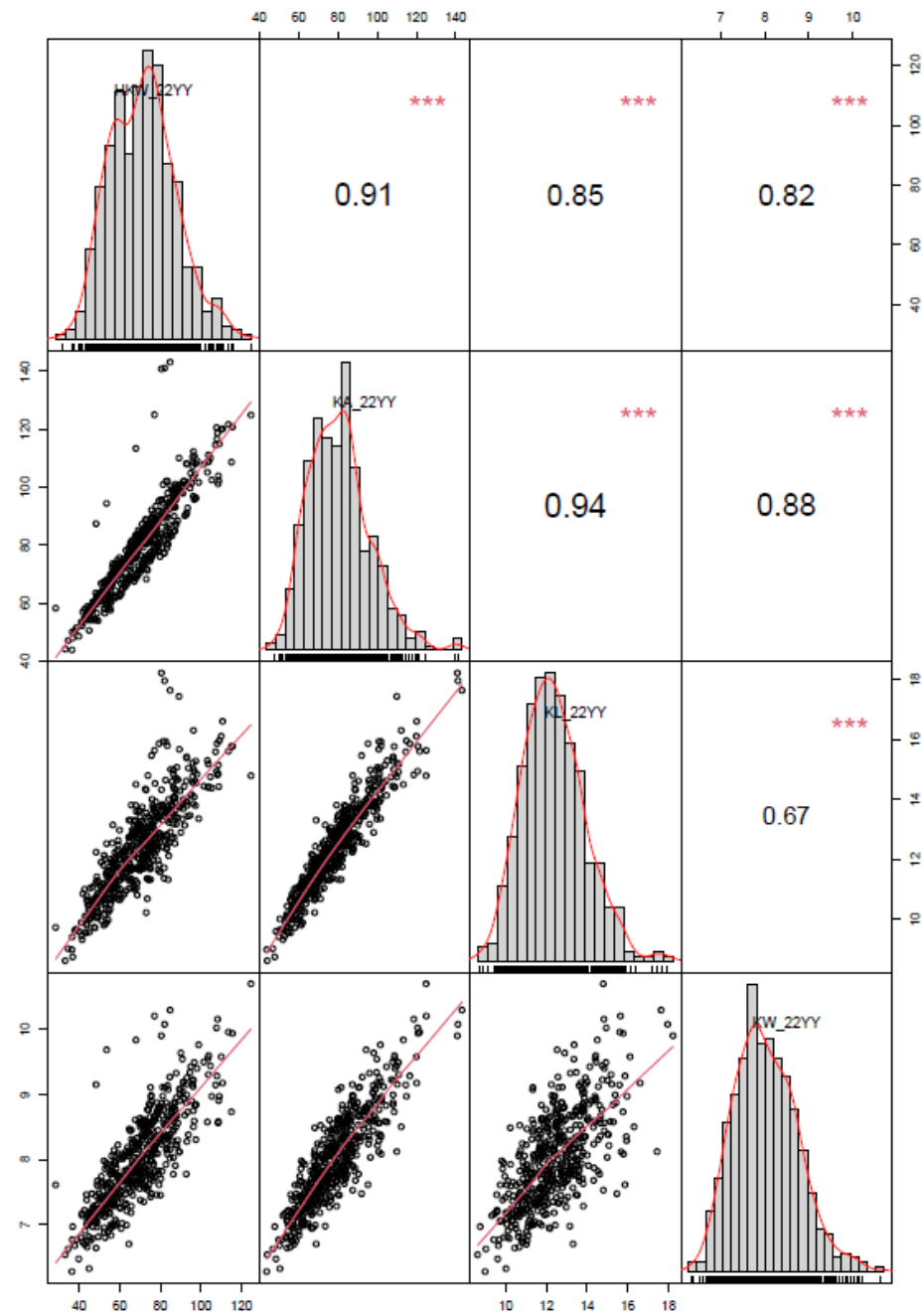

**Fig. S5** Correlation between HKW, KA, KL and KW in Yuanyang 2022.

Supplement: Supplementary file 6 — Additional file 6: Fig S5. Correlation between HKW, KA, KL and KW in Yuanyang 2022. [file 12864_2023_9568_MOESM6_ESM.pdf]

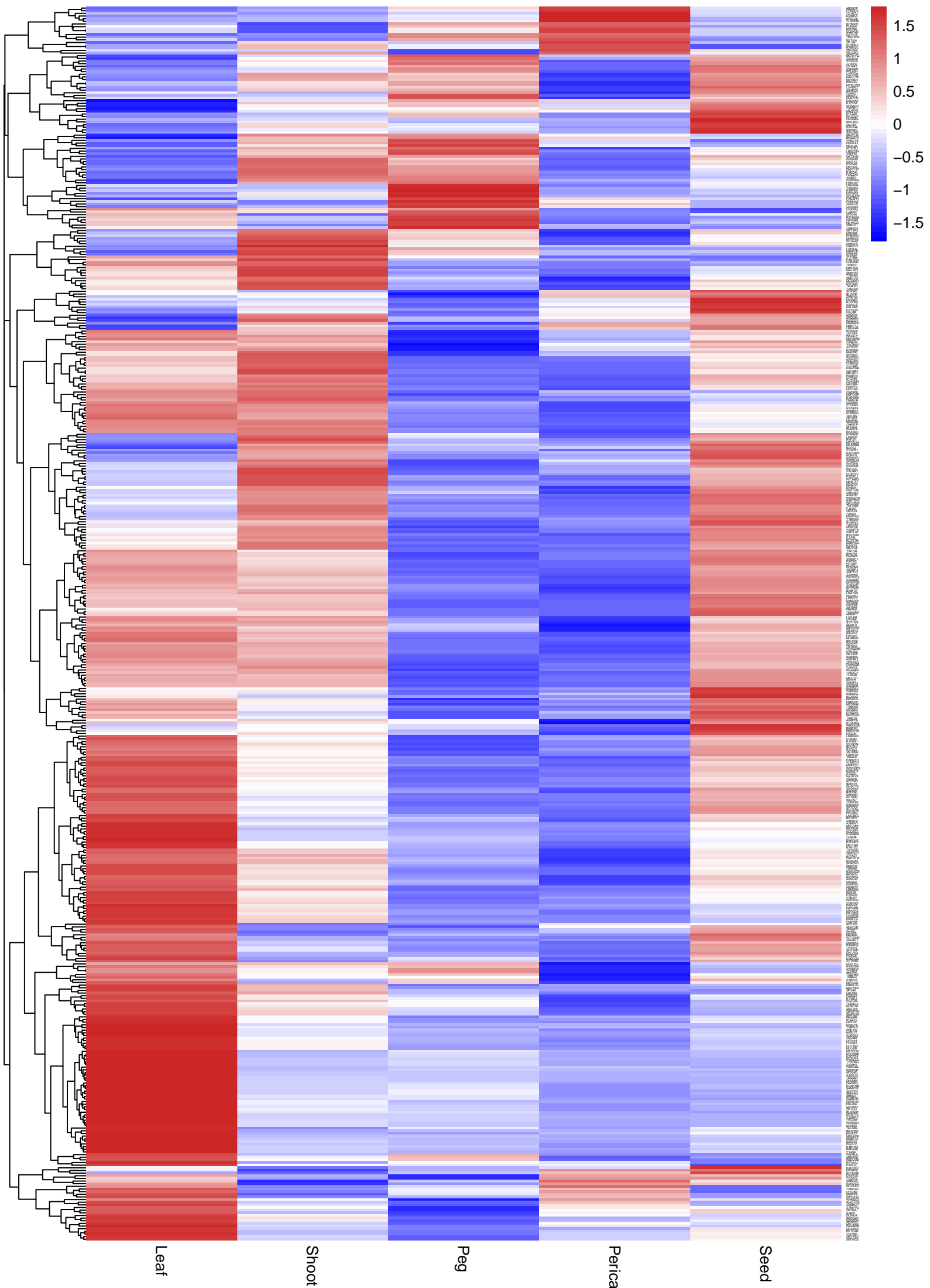

Supplement: Supplementary file 10 — Additional file 10: Fig S9. Heatmap of the differentially expressed PPR genes across five different tissues of Tifrunner including leaf, shoot, peg, pericarp, and seed. [file 12864_2023_9568_MOESM10_ESM.pdf]
