# Supplementary material for: Identification of QTL for kernel weight and size and analysis of the pentatricopeptide repeat (PPR) gene family in cultivated peanut (Arachis hypogaea L.)
Source: BMC Genomics. 2023 Aug 28;24:495. doi: 10.1186/s12864-023-09568-y (PMC10463326; doi:10.1186/s12864-023-09568-y)
Supplement: Supplementary file 7 — Additional file 7: Fig S6. Distribution of the Pentatricopeptide Repeat (PPR) gene family in the Arachis hypogaea cv Tifrunner genome. [file 12864_2023_9568_MOESM7_ESM.pdf]

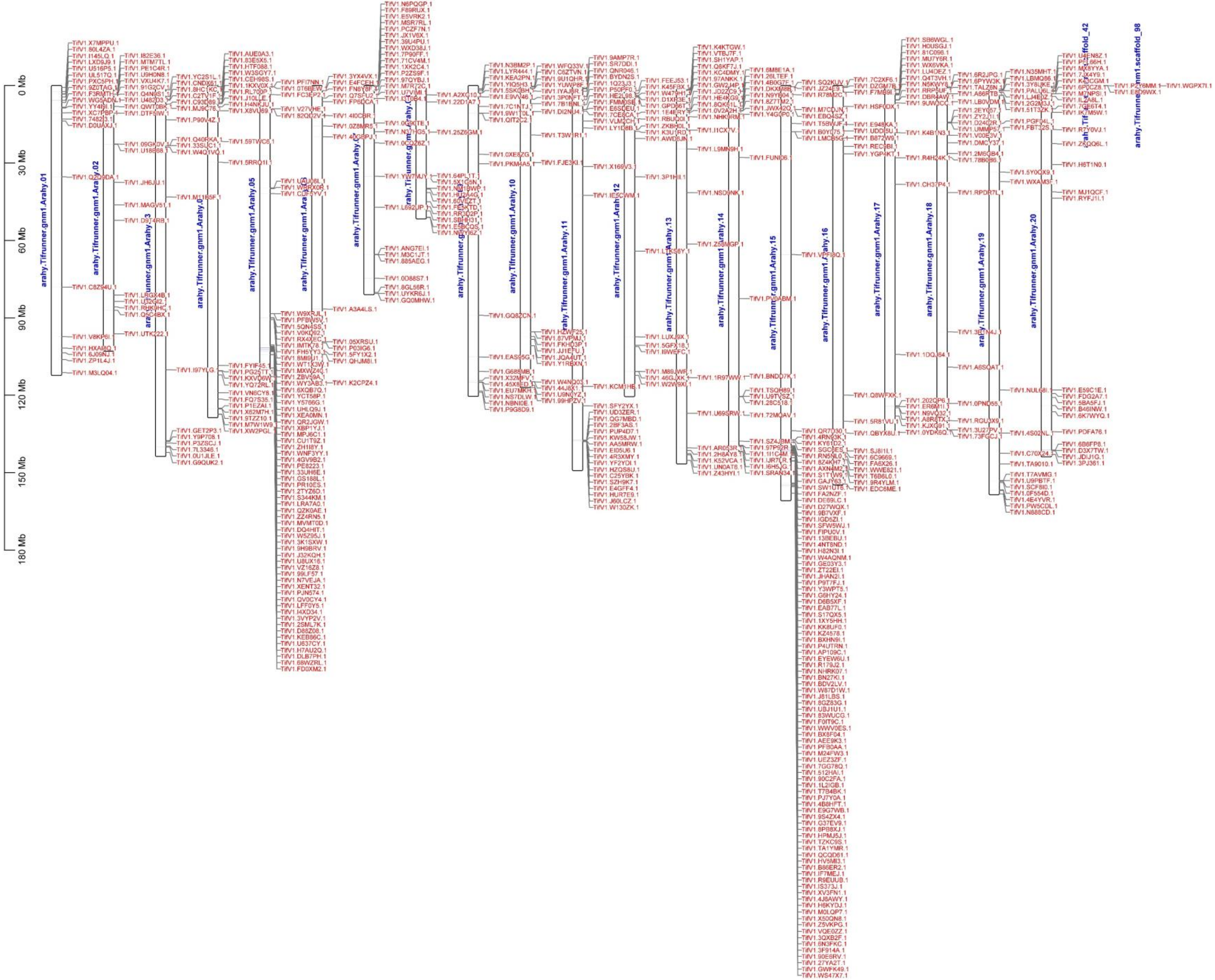

Fig. S6 Distribution of the *Pentatricopeptide Repeat* (*PPR*) gene family in the *Arachis hypogaea* cv Tifrunner genome.
