# Supplementary material for: Identification of QTL for kernel weight and size and analysis of the pentatricopeptide repeat (PPR) gene family in cultivated peanut (Arachis hypogaea L.)
Source: BMC Genomics. 2023 Aug 28;24:495. doi: 10.1186/s12864-023-09568-y (PMC10463326; doi:10.1186/s12864-023-09568-y)
Supplement: Supplementary file 11 — Additional file 11: Fig S10. Heatmap of the differentially expressed PPR genes across four different stages of seed development in Tifrunner. [file 12864_2023_9568_MOESM11_ESM.pdf]

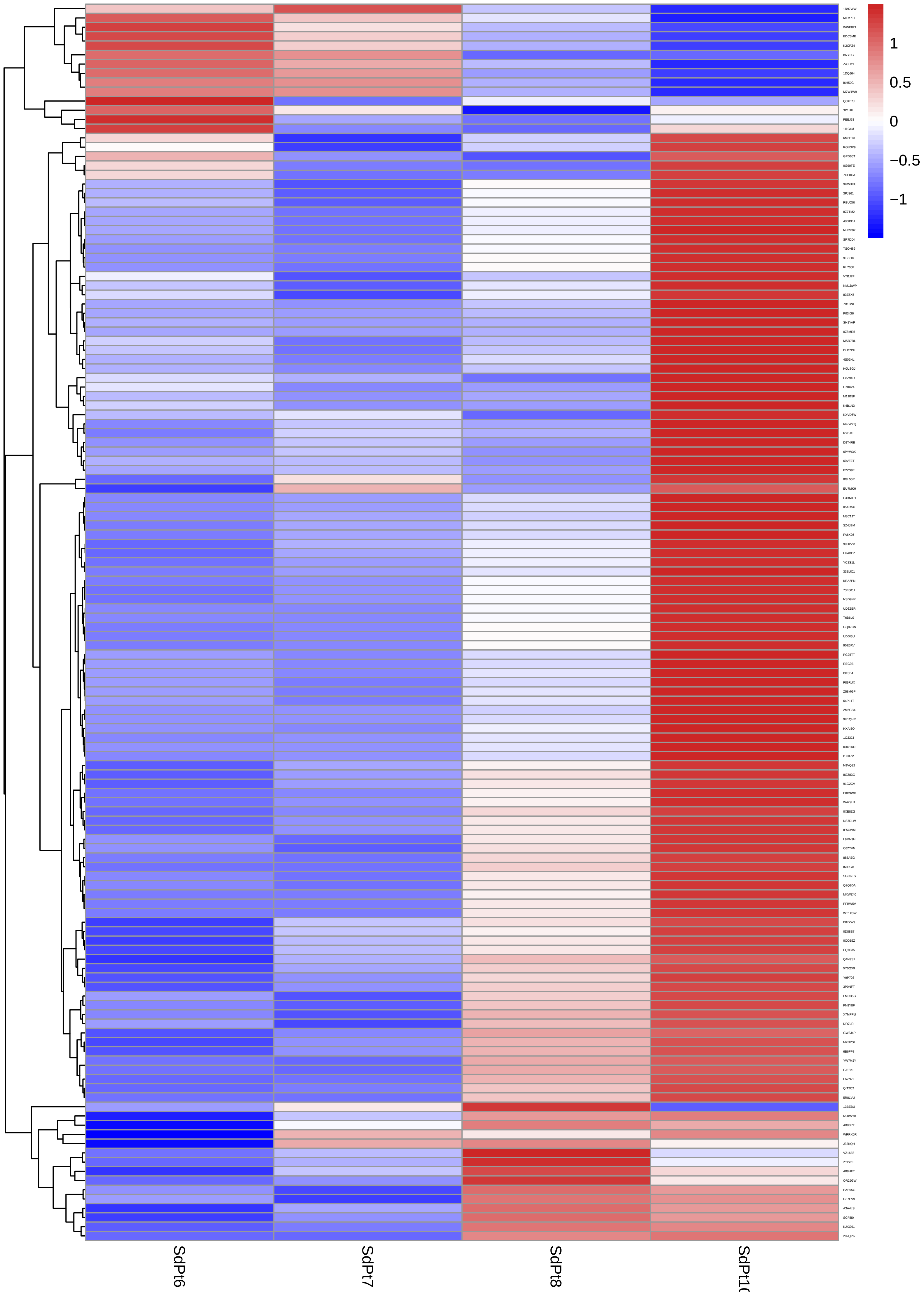

Fig. S10 Heatmap of the differentially expressed PPR genes across four different stages of seed development in Tifrunner.
