# Supplementary material for: Identification of QTL for kernel weight and size and analysis of the pentatricopeptide repeat (PPR) gene family in cultivated peanut (Arachis hypogaea L.)
Source: BMC Genomics. 2023 Aug 28;24:495. doi: 10.1186/s12864-023-09568-y (PMC10463326; doi:10.1186/s12864-023-09568-y)
Supplement: Supplementary file 8 — Additional file 8: Fig S7. Gene structure of the Pentatricopeptide Repeat (PPR) genes in the cultivated peanut (Arachis hypogaea L.). [file 12864_2023_9568_MOESM8_ESM.pdf]

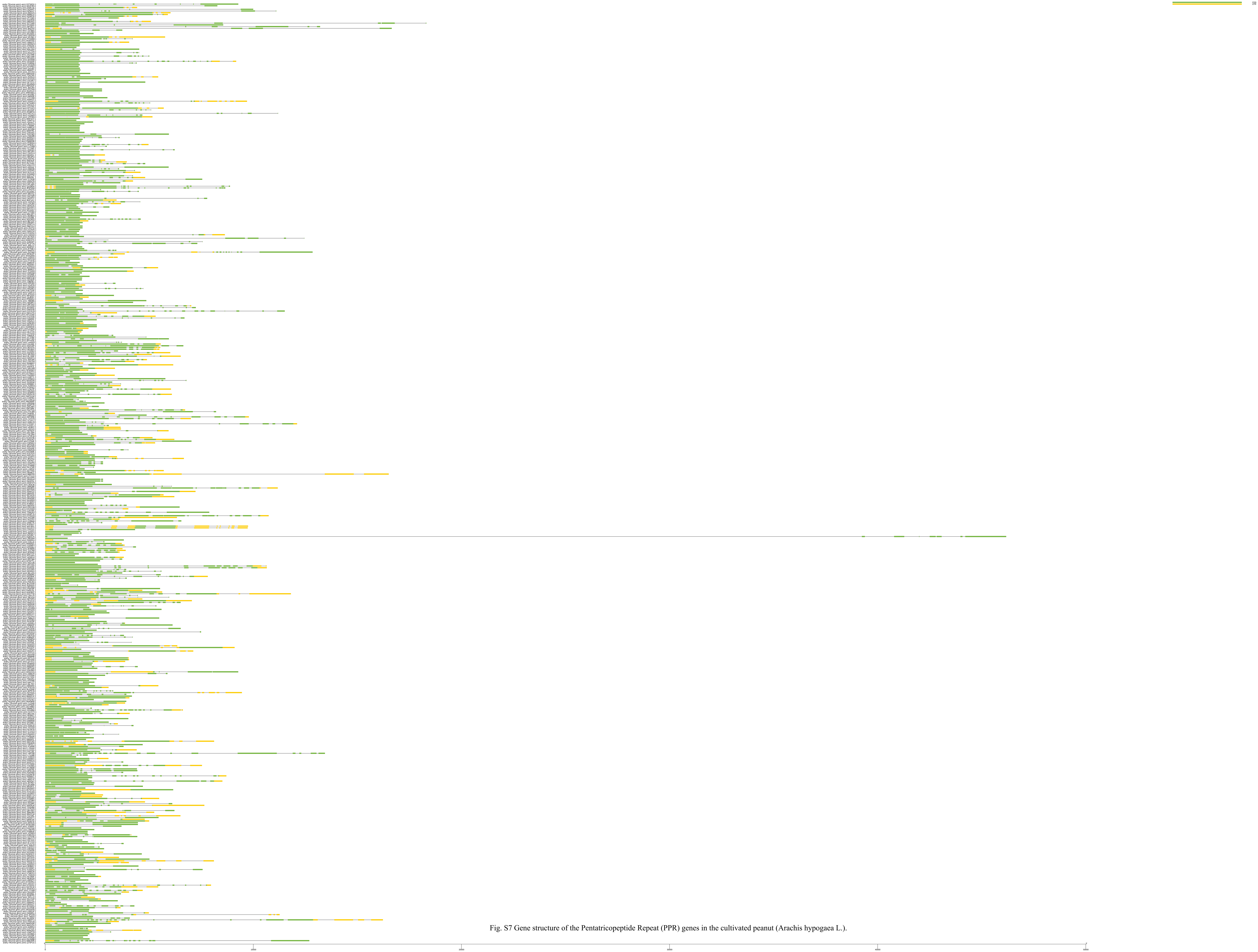

Fig. S7 Gene structure of the Pentatricopeptide Repeat (PPR) genes in the cultivated peanut (Arachis hypogaea L.).
